# Advancing crush syndrome management: the potent role of Sodium zirconium cyclosilicate in early hyperkalemia intervention and survival enhancement in a rat model

**DOI:** 10.3389/fphar.2024.1381954

**Published:** 2024-05-13

**Authors:** Duo Li, Yan Zhang, Yuansen Chen, Bofan Yang, Jianwen Chen, Jie Shi, Xiaoqin Guo, Yanqing Liu, Li Zhang, Qi Lv, Haojun Fan

**Affiliations:** ^1^ Institute of Disaster and Emergency Medicine, Tianjin University, Tianjin, China; ^2^ Wenzhou Safety (Emergency) Institute, Tianjin University, Wenzhou, China; ^3^ Department of Nephrology, National Key Laboratory of Kidney Diseases, National Clinical Research Center for Kidney Diseases, Military Logistics Research Key Laboratory of Field Disease Treatment, Beijing Key Laboratory of Kidney Disease Research, First Medical Center of Chinese PLA General Hospital, Beijing, China

**Keywords:** crush syndrome, Sodium zirconium cyclosilicate, hyperkalemia, rat model, survival rate

## Abstract

**Background:** Crush Syndrome (CS), a severe trauma resulting from prolonged muscle compression, is commonly seen in large-scale disasters such as earthquakes. It not only causes localized tissue damage but also triggers electrolyte imbalances, particularly hyperkalemia, increasing the risk of early mortality. This study aims to assess the early intervention effects of Sodium Zirconium Cyclosilicate (SZC) on hyperkalemia in rat CS model.

**Methods:** A rat CS model was established using a self-developed multi-channel intelligent small-animal crush injury platform. Rats in the experimental groups were treated with varying doses of SZC before compression and immediately post-decompression. The efficacy of SZC was evaluated by continuous monitoring of blood potassium levels and survival rates. Serum creatinine (Cre) and blood urea nitrogen (BUN) levels were analyzed, and renal damage was assessed through histopathological examination.

**Results:** SZC treatment significantly reduced blood potassium levels and improved survival rates in rats. Compared to the placebo group, the SZC-treated rats showed a significant decrease in blood potassium levels at 6 and 12 h post-decompression, maintaining lower levels at 24 h. Biochemical analysis indicated no significant impact of SZC on renal function, with no notable differences in Cre and BUN levels between groups. Histopathological findings revealed similar levels of renal damage in both groups.

**Conclusion:** SZC demonstrates significant early intervention effects on hyperkalemia in a rat model of crush injury, effectively improving survival rates without adverse effects on renal function. These results provide a new strategic direction for the clinical treatment of Crush Syndrome and lay the foundation for future clinical applications.

## Introduction

Crush Syndrome, also known as traumatic rhabdomyolysis, is a grave trauma typically linked with large-scale disasters like earthquakes, resulting from prolonged compression of skeletal muscles (Zhang et al., 2017; Candela et al., 2020). Beyond direct trauma, Crush Syndrome is the second leading cause of death in earthquake-related injuries (Sever and Vanholder, 2013). Severe crush injuries not only cause localized tissue damage but often trigger systemic physiological responses (Gupta et al., 2021). Among these, the most clinically significant early issue is electrolyte imbalance, particularly hyperkalemia (Fan et al., 2016). The destruction of muscle and other tissues leads to a substantial release of potassium ions from cells into the bloodstream, causing elevated blood potassium levels (Bansal and Pergola, 2020). This acute hyperkalemia can lead to arrhythmias, impaired myocardial function, and even cardiac arrest, a critical factor in the early mortality of patients with crush injuries (Linde et al., 2019; Bansal and Pergola, 2020; Reilly et al., 2020). Timely and effective potassium-lowering treatments are essential for improving patient survival rates. However, in scenarios of mass casualties, resource constraints, and overwhelmed medical personnel, the limitations of current mainstream early treatments such as fluid resuscitation, forced diuresis, and renal replacement therapy become apparent (Kosiborod et al., 2015). Safe and effective drugs to reduce on-site mortality in crush syndrome are clinically vacant (Fan et al., 2016). In this context, exploring novel therapeutic approaches, such as the application of innovative drugs, may provide new strategies for managing post-crush injury hyperkalemia, thereby improving patient outcomes and reducing on-site mortality.

Sodium Zirconium Cyclosilicate (SZC) is an oral, non-absorbable intestinal potassium binder. It consists of inorganic crystals that are highly compatible in diameter with potassium ions, binding to potassium with a force 25 times greater than that of other cations (Stavros et al., 2014). SZC operates in the gastrointestinal tract by exchanging with potassium ions, thus reducing potassium levels in the blood (Amin et al., 2019). It has shown significant effectiveness in controlling hyperkalemia. In multiple clinical trials, SZC has been proven to effectively lower blood potassium levels in patients with hyperkalemia and is generally considered safe with good tolerability (Kosiborod et al., 2014; Kashihara et al., 2021; Zhang et al., 2021; Qu et al., 2023). Its universality is evident in real-world studies across different ethnic groups, where patients receiving SZC for the treatment or prevention of hyperkalemia experienced a significant reduction in mortality rates (Qu et al., 2023; Onogi et al., 2024). SZC not only shows significant efficacy in the treatment of hyperkalemia in adults but is also safe and effective for acute and chronic hyperkalemia in children (Khandelwal et al., 2024). Additionally, studies indicate that in patients with heart failure, SZC can help maintain normal potassium levels, allowing the continued use of medications that might cause hyperkalemia, such as renin-angiotensin-aldosterone system (RAAS) blockers (Anker et al., 2015; Butler et al., 2019; Abrignani et al., 2024; Kimura et al., 2024). In patients with Chronic Kidney Disease (CKD), SZC corrects hyperkalemia and maintains normokalemia among outpatients, regardless of the stage of CKD (Roger et al., 2021). Moreover, SZC effectively reduces pre-dialysis hyperkalemia in patients with End-Stage Renal Disease (ESRD) (Fishbane et al., 2019). These findings suggest the potential of SZC to manage hyperkalemia in various clinical settings.

Although existing literature suggests that SZC is effective in patients with chronic conditions, its application and efficacy in acute trauma scenarios, particularly in crush injury models, have not yet been reported. Given that crush injuries often lead to acute hyperkalemia, exploring the use and effects of SZC in this specific clinical context is crucial. This study aims to investigate the early intervention effects of SZC on hyperkalemia in a rat model of crush injury. The goal is to fill this knowledge gap, providing more evidence and treatment options for the clinical management of future crush injury patients.

## Materials and methods

### Animals and CS model construction

Sprague-Dawley (SD) rats (280–300 g) were purchased from Beijing Huafukang Biotechnology Company. These rats were housed in a specific pathogen-free facility under a 12-h light/12-h dark cycle with free access to food and water. Rat CS model were constructed by self-developed multi-channel intelligent small-animal crush injury platform according to methods previously described (Li et al., 2023).

### Dynamic monitoring

**FIGURE 1:**
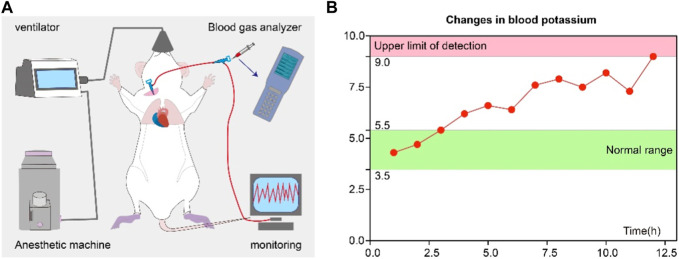
Continuous monitoring of blood potassium changes in a rat post-decompression. **(A)** Diagram illustrating the continuous monitoring of serum potassium and the configuration of the monitoring equipment. **(B)** Serum potassium change over time curve. The red line represents the serum potassium levels, the green area indicates the normal range for serum potassium, and the pink area indicates the detection limit of the iSTAT handheld blood analyzer.

A rat was randomly selected post-decompression and anesthetized with 2.5% isoflurane before being secured on the machine operating table. A disposable venous indwelling needle was used for carotid artery cannulation, which was then connected to a transducer. Blood pressure (BP) and heart rate (HR) were continuously measured using Biopak Systems data acquisition system (Santa Barbara, CA). Arterial blood samples were taken every hour using a 1 mL syringe through the indwelling needle. Blood potassium analysis was performed using an iSTAT handheld blood analyzer (Abbott, United States) (Figure 1A).

### SZC treatment

**FIGURE 2:**
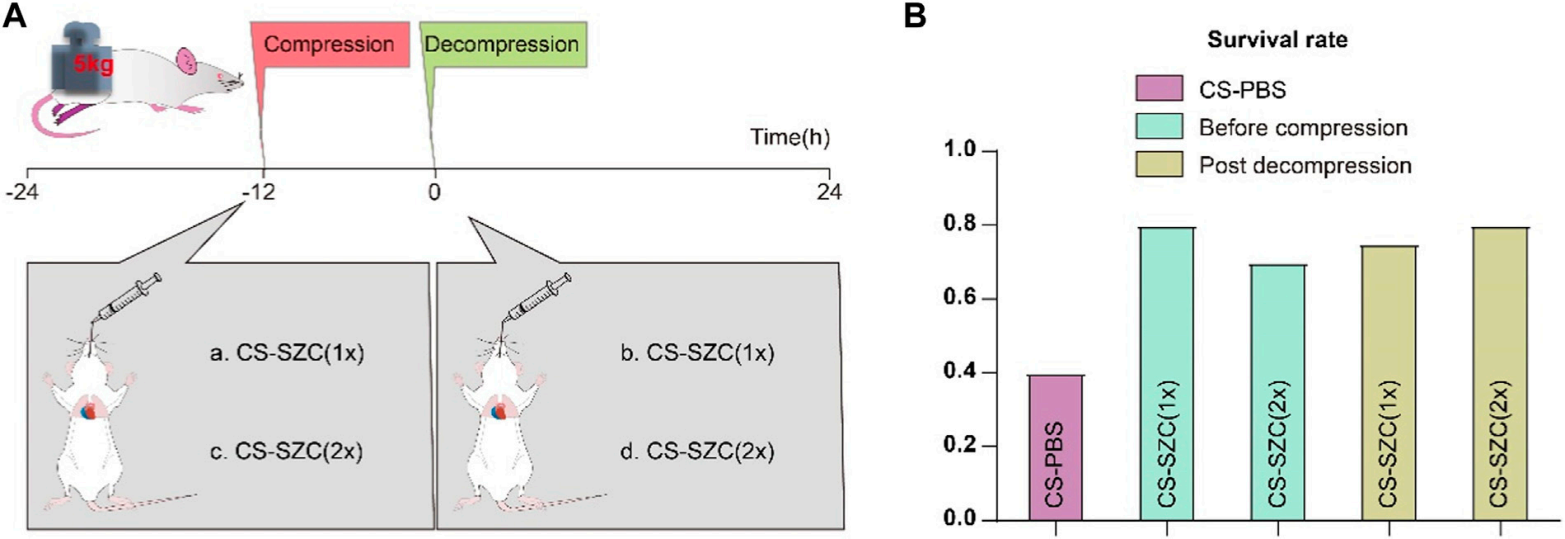
The Effect of SZC Treatment on Survival Rates in rat CS model. **(A)** Experimental Timeline and Drug Intervention Protocol. The experimental groups were divided into four subgroups: a. rats administered a standard dose of SZC before compression; b. rats administered a double dose of SZC before compression; c. rats administered a standard dose of SZC immediately post decompression; d. rats administered a double dose of SZC immediately post decompression. **(B)** Comparison of Survival Rates. Colour of squares represent the timing of SZC (or PBS) treatment, purple squares represent the CS-PBS group, green squares represent before compression, yellow squares represent post decompression group.

All rats received an oral suspension of SZC (AstraZeneca) via gavage. The drug treatment groups were divided into four categories as shown in Figure 2A. Group a: Administered standard dose of SZC via gavage before compression. Group b: Administered double the standard dose of SZC via gavage before compression. Group c: Administered standard dose of SZC via gavage immediately after decompression. Group d: Administered double the standard dose of SZC via gavage immediately after decompression. The standard dose of SZC was calculated to be 900 mg/kg (According to the medication guide and the standard for dose conversion between rats and humans).

### Survival of the CS model

We observed the differences in survival times among rats in each group. The observation endpoint was 24 h post-decompression.

### Blood sample analysis

Blood samples were collected from the abdominal vena cava at specified time intervals. To separate the serum from the plasma, the blood samples were allowed to stand for a minimum of 30 min. Following this, centrifugation was performed at 3,000 rpm for 15 min at 4°C. The levels of serum potassium, Cre and BUN were measured by using an automated multi-parameter biochemical analyzer (Olympus, Japan).

### Histopathological analysis

After the experiment, the sample was immediately collected, and the kidney was transferred into 4% paraformaldehyde at 4 °C for fixation for 24 h. Subsequently, the sample underwent paraffin embedding and was sectioned into 5-µm-thick. Then periodic acid-Schiff (PAS) staining was performed to assess tubular injury.

### Statistical analysis

Data are presented as the mean ± SEM. Analysis was conducted with GraphPad Prism (version 9.0.0). If the data are normally distributed, an independent sample t-test is used for statistical analysis; if the data are not normally distributed, a two-sample independent rank-sum test is applied. Statistical significance was set at *p* < 0.05.

## Results

### Dynamic changes of blood potassium in a rat post decompression

In our continuous monitoring of blood potassium in rats post-decompression, we observed the characteristic changes in potassium ion concentration in the blood. Figure 1B reveals the trend of serum potassium changes from the moment of decompression to the 12th hour. Preliminary data indicate that in the early stages post-decompression, the rats’ serum potassium levels remained within the normal range (3.5–5.5 mmol/L). However, as time progressed, the levels of serum potassium showed an increasing trend, exceeding the normal range starting at the 3rd hour and reaching the upper limit of detection at the 12th hour. In the figure, the normal range of serum potassium values is represented by a green area, while the pink area indicates the detection limit of the iSTAT handheld blood analyzer.

### Survival rate analysis

We evaluated the effects of different doses of SZC and the timing of its administration on rat survival rates in CS model. The results showed that the survival rate in the placebo group (which did not receive SZC treatment) was 40%. There was a significant increase in survival rates in all groups that received the drug intervention, although the differences in survival rates among these intervention groups were relatively small. Specifically, the survival rates for rats administered a standard dose of SZC (Group a) and a double dose of SZC (Group b) prior to compression were 80% and 70%, respectively. For rats given a standard dose of SZC (Group c) and a double dose of SZC (Group d) immediately post decompression, the survival rates were 75% and 80%, respectively (Figure 2B).

### Biochemical and pathological changes

**FIGURE 3:**
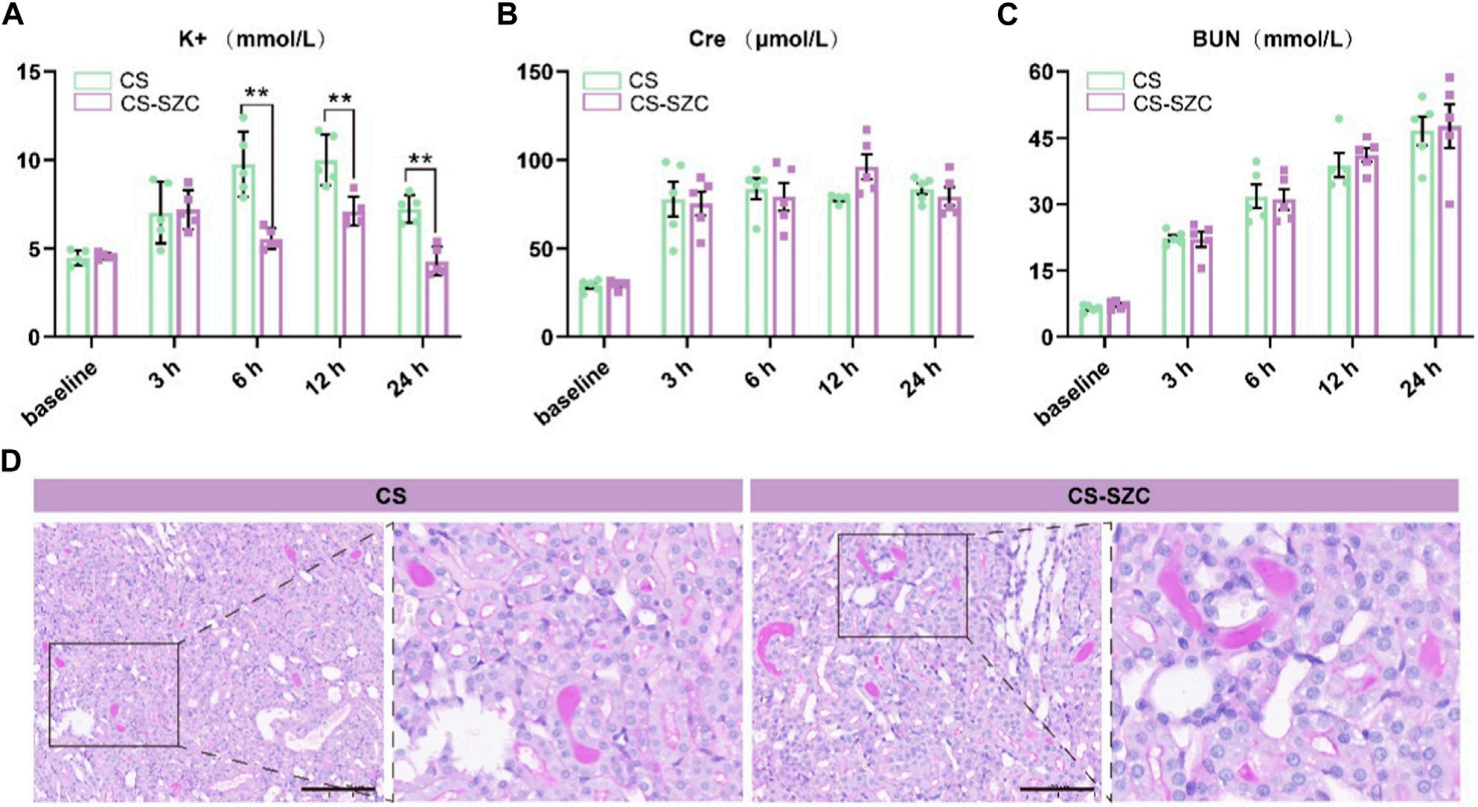
Comparison of the effects of standard-dose SZC treatment on biochemical indices and Renal Function in rat CS model. **(A)** Serum potassium levels; **(B)** Serum Cre levels; **(C)** Serum BUN levels; green squares represent the CS group, red squares represent the CS-SZC group. n = 5, *p < 0.05, **p < 0.01, ***p < 0.001, ns represents no statistical significance. **(D)** Representative images of PAS stain of renal cortex in different groups post decompression 24 h, Scale bars: 100 μm.

The changes in serum potassium levels at various time points post-decompression are shown in Figure 3A. At 6 h and 12 h post-decompression, the serum potassium levels in the SZC treatment group were significantly lower compared to the placebo group. At 24 h, serum potassium levels remained lower, and although they had risen compared to the baseline, there was still a significant difference compared to the placebo group. The analysis of Cre levels showed no difference between the two groups at any time point (Figure 3B). Furthermore, there were no significant differences in BUN levels between the SZC treatment and placebo groups (Figure 3C). In both groups of rats, the kidney PAS staining revealed distinct protein casts and tubular dilation, as well as desquamation of the brush border, with no substantial necrosis of tubular epithelial cells observed (Figure 3D). The level of kidney pathological damage was comparable between the two groups. This finding is consistent with the biochemical results, indicating that SZC does not have a significant impact on renal function. The intra-group comparative analysis of the SZC intervention group showed that the overall trend of these three indicators was consistent with that of the placebo group.

## Discussion

This study is the first to implement continuous dynamic monitoring of blood potassium in rat CS model of crush injury, filling a research gap in this field. In our continuous monitoring of blood potassium levels post-decompression, we observed characteristic changes in the concentration of potassium ions in the blood. In the early stages post-decompression, the rats’ potassium levels remained within the normal range (3.5–5.5 mmol/L). However, over time, potassium levels showed an upward trend, exceeding the normal range starting from the 3rd hour and reaching the upper detection limit by the 12th hour. The overall upward trend indirectly reflects the early onset and progressive aggravation of muscle cell lysis and necrosis. Although the early values remained within the normal range and did not reach a critical level requiring immediate intervention, they still warrant our close attention. This data also underscores the importance of continuous monitoring of blood potassium levels in the management of crush injuries. Our findings provide new insights into the temporal sequence of physiological changes following crush injuries and may guide the timing of emergency measures.

Early severe hyperkalemia (serum potassium level ≥6 mmol/L) can cause fatal cardiac arrhythmias and is a major factor in on-site mortality (Thomsen et al., 2018; Acelajado et al., 2019). Timely correction of post-decompression hyperkalemia in casualties is essential. However, due to chaotic environments and limited resources at disaster sites, casualties often do not receive timely treatment (Orsini et al., 2020). Existing therapies have significant limitations, such as the short-term efficacy of intravenous calcium, bicarbonate, and polarizing solutions, which are uncertain and cannot rapidly remove excess potassium; exchange resins have poor safety; and emergency hemodialysis is invasive and costly (Kosiborod et al., 2015; Thomas et al., 2016; Himmelfarb et al., 2020; Luyckx et al., 2021). The emergence of the new oral potassium-lowering drug SZC has brought hope to this dilemma (Kosiborod et al., 2014). This study explored the impact of SZC on the early mortality rate of crush injuries in a rat model of gastric lavage intervention. The results showed that the survival rate in the placebo group (which did not receive SZC treatment) was 40%. In all drug intervention groups, the survival rate was significantly improved, but the differences in survival rates between these intervention groups were relatively small. Specifically, the survival rates of rats given a standard dose of SZC (Group a) and a double dose of SZC (Group b) before compression were 80% and 70%, respectively. For rats given a standard dose of SZC (Group c) and a double dose of SZC (Group d) immediately post-decompression, the survival rates were 75% and 80%, respectively. Both standard and double doses of SZC have shown effectiveness in controlling mortality rates in CS rats, with no significant dose-response relationship observed. This aligns with the findings of William Shockey et al., whose study demonstrated that a single 10 g dose of SZC significantly reduced serum potassium levels across all patient subgroups, without any additional benefits from adjunctive potassium-lowering therapies (Shockey et al., 2024). Another study also indicated that a single 10 g dose of SZC per day was more effective in normalizing serum potassium levels compared to administering 10 g three times a day (Lewis et al., 2024). Therefore, a single 10 g dose of SZC may be a reasonable treatment option. Following immediate post-decompression administration, standard-dose SZC treatment significantly reduced blood potassium levels from 3 to 12 h compared to baseline. Although there was a subsequent rise in blood potassium levels at 24 h, they remained significantly lower than those in the placebo group. This indicates that SZC can effectively reduce the risk of early hyperkalemia after crush injury. This is crucial for resource allocation in mass casualty events, as using higher doses of SZC may not translate into additional survival benefits and may lead to resource waste.

However, it is worth noting that although SZC can control blood potassium levels, no impact of SZC on renal function was found in biochemical and renal tissue pathological analysis; Cre and BUN levels did not change significantly at any time point compared to the control group. These results further confirm the potential of SZC as a treatment for crush injuries, effectively controlling blood potassium levels without affecting renal function. The study of Li et al. also indicates that SZC has a minimal impact on renal function. There were no statistically significant differences in serum Cre levels among the three treatment groups (*p* > 0.05) (Li et al., 2024). Current models of crush injury-related renal injury often use glycerol for intramuscular injection to construct a model of rhabdomyolysis as a substitute (Belliere et al., 2015; Rosenkrans et al., 2020). The advantage of this model is that it can cause severe kidney damage with a lower mortality rate, but the downside is that it cannot truly simulate the crush injury at the disaster site. The main reason for not being able to create a more severe kidney damage model through compression is that animals usually die of early hyperkalemia. SZC’s effective control of blood potassium without affecting renal function significantly reducing mortality may provide a new method to help build a more severe kidney damage model.

In summary, our results show that SZC has significant early intervention effects on hyperkalemia in a rat model of crush injury, effectively improving survival rates without adversely affecting renal function. In the unique clinical scenario of crush injury, SZC demonstrates rapid and significant potassium-lowering effects, which may help reduce the early mortality rate of casualties at disaster sites. Its potassium-lowering efficacy and safety may make it a valuable addition to existing CS treatment methods. Future research should further explore the application of SZC in crush injury scenarios and its impact on overall rescue strategies, including resource allocation, drug storage, and emergency training. Our study paves the way for further research in this field.

## Data Availability

The original contributions presented in the study are included in the article/Supplementary material, further inquiries can be directed to the corresponding authors.
